# Associations of SARS-CoV-2 PCR positivity with clinical symptoms and race/ethnicity: The household transmission study

**DOI:** 10.1371/journal.pone.0332819

**Published:** 2025-09-30

**Authors:** Sara H. Goodman, Jonathan Altamirano, Julianne Burns, Clea S. Sarnquist, Jennifer S. Read, Yvonne Maldonado

**Affiliations:** 1 Department of Pediatrics, Division of Infectious Diseases, Stanford University School of Medicine, Stanford, California, United States of America; 2 Department of Epidemiology and Population Health, Stanford University School of Medicine, Stanford, California, United States of America; Makerere University, UGANDA

## Abstract

**Objective:**

The objective of this study was to identify demographic and clinical factors associated with SARS-CoV-2 infection among household contacts (HCs) following exposure to a confirmed case.

**Methods:**

We analyzed an existing case-ascertained prospective cohort study of 43 HCs of SARS-CoV-2-positive index cases recruited from Stanford Health Care between March 2020 and June 2022. Participants self-collected nasal swabs daily for up to 21 days for reverse transcription polymerase chain reaction (RT-PCR) testing and reported symptoms in daily diaries. Using Cox proportional hazards models, we assessed associations between participant characteristics over time to first positive PCR result.

**Results:**

We found that symptomatology and race/ethnicity were independently associated with increased infection risk. In multivariable analysis, participants with systemic symptoms had a higher likelihood of testing positive (adjusted hazard ratio [aHR]=2.62; 95% confidence interval [CI]: 1.38–6.55). Additionally, identifying as a racial/ethnic minority had a greater risk of a positive test (aHR = 2.55 for systemic symptoms, 2.43 for any respiratory symptoms, and 2.40 for upper respiratory symptoms) compared to white patients.

**Conclusion:**

These findings underscore the importance of symptom-based surveillance and highlight ongoing racial and ethnic disparities in SARS-CoV-2 transmission risk. This study also demonstrates the feasibility of longitudinal, self-administered testing and offers a scalable model for investigating transmission dynamics of respiratory viruses in community settings.

## Introduction

SARS-CoV-2 remains a public health concern five years after the initial outbreak. Households play a significant role in SARS-CoV-2 transmission, and understanding risk factors for household transmission is a crucial step toward mitigation, disease prevention, and infection control. Multiple factors have been associated with increased household transmission, including higher viral load and under-vaccination (making participants more susceptible to viral infection) [1,2]. We utilized data from a unique prospective cohort study examining household transmission of SARS-CoV-2 to analyze potential associations between a positive reverse transcription polymerase chain reaction (PCR) test and demographic and clinical characteristics and symptomatology among household contacts (HCs) of SARS-CoV-2-infected individuals (index cases, ICs).

## Materials and methods

The Household Transmission Study (HTS) was a prospective cohort study that enrolled outpatient ICs evaluated in the Stanford University Medical Center Emergency Department or at local testing centers within Stanford Health Care with a positive SARS-CoV-2 PCR assay (Panther Fusion SARS-CoV-2 Assay) from a nasopharyngeal swab obtained between March 25, 2020 and June 30, 2022 [3,4]. At the household enrollment visit, a structured questionnaire in REDCap was administered to collect sociodemographic and health data from ICs and HCs [4]. Participants self-collected nasal swabs (CLASSIQ Swabs; Copan Diagnostics) for PCR testing for at least 21 days, or until every HC had seven consecutive negative tests, whichever came first. PCR assays were deemed positive if the Ct value was below the Stanford Clinical Laboratory cut-off of 45 [3–5]. SARS-CoV-2-related symptoms were self-reported by participants in daily diaries throughout the study. The researchers accessed the data between March 25,2020 and November 30, 2024.

The Stanford University Administrative Panel on Human Subjects in Medical Research approved the research on March 25, 2020 (Protocol #55479), and all participants provided written informed consent. The study population for this analysis comprised all HCs with one or more detectable SARS-CoV-2 PCR assays with at least one negative PCR assay before a positive assay.

Data were analyzed using R and Stata 18 statistical software. The dependent variable was the first positive SARS-CoV-2 PCR test result. Independent variables included age (as continuous), sex assigned at birth, and race/ethnicity categorized as white, Asian, and racial/ethnic minorities (including African-American, Hispanic/Latine, other races, one or more races, mixed races, American Indian/Alaska Native, Native Hawaiian/Pacific Islander). We used this categorization to group minority participants as informed by the literature as one proxy for social disparities addressed in studies showing patients who identified as African-American/Latine were more likely to have adverse SARS-CoV-2 outcomes compared to patients who identified as white or Asian [6,7]. We also combined these categories due to the small cell sizes if analyzed separately. We performed bivariable Cox proportional hazard analyses using the Efron method for ties, with each covariate regressed on PCR positivity to determine covariates for multivariable modeling. We chose our independent variables to measure time to PCR positivity.

## Results

The HTS enrolled 191 participants, of which 68 were ICs and 123 were HCs. Of the 123 HCs, 67 never had a positive PCR assay during the study period. Of the remaining 56 HCs, 43 had one or more negative PCR assay result(s)before a positive one. Thus, the study population for this analysis comprised 43 HCs (Table 1). The median total number of PCR tests per HC was 21 (interquartile range (IQR): 21 (19–3 1)). The median number of positive PCR tests per HC was 5 (IQR = 1–9). The median time to the first negative PCR assay result after at least one positive result was 12 days (IQR 9–18). The median number of days testing positive was 20 (IQR 14–30). The median time to the highest estimated viral load (lowest Ct value) was six days (IQR 3–8) (S1 Fig).

**Table 1:** Characteristics of the study population on the day of a first positive PCR assay (N = 43).

Part A – Categorical variables		
		N (%)
**Race**		
Asian		10 (23%)
Racial/ethnic minority		10 (23%)
White		23 (53%)
**Sex assigned at birth**		
Male		21 (49%)
Female		22 (51%)
**Education**		
Less than primary school completion		5 (12%)
More than primary school less than secondary school completion		4 (11%)
Completed secondary school		0 (0%)
College/university/technical college		26 (74%)
*Missing*		*8 (19%)*
**Occupation**		
Science/technology		11 (26%)
Medicine/healthcare		4 (9%)
Service/education		8 (19%)
Business/finance		5 (12%)
Retired		4 (9%)
Other		2 (5%)
*Missing*		*9 (21%)*
**Self-reported symptoms on the date of the first positive PCR test?** ^**a**^		
**Any symptoms**		
Yes		25 (58%)
No		18 (42%)
**Systemic symptoms**		
Yes		16 (37%)
No		27 (63%)
**Any respiratory symptoms**		
Yes		15 (35%)
No		28 (65%)
**Upper respiratory symptoms**		
Yes		12 (28%)
No		31 (72%)
**Lower respiratory symptoms**		
Yes		4 (9%)
No		39 (91%)
**Part B – Continuous variables**		
**Continuous Variables**	**Mean (SD)**	**Median (IQR)**
Age (years)	36.63 (19.99)	39.00 (19.00–52.00)
Household size (number of people in household)	3.26 (0.82)	3.00 (3.00–40.00)
Total number of PCR tests per participant	26.53 (11.68)	21.00 (19.00–31.00)

^a^**Systemic:** Myalgias (2), fatigue/malaise (10), anosmia (5), ageusia (4), loss of appetite (1), fussiness (in an infant) (1)

**Any Respiratory:** Sore throat (9), rhinorrhea (6), nasal congestion (2), shortness of breath (1), chest pain (1), cough (2)

**Upper respiratory:** Sore throat (9), rhinorrhea (6), nasal congestion (2)

**Lower respiratory:** Shortness of breath (1), chest pain (1), cough (2)

In bivariable Cox proportional hazard modeling (S1 Table), identifying as a racial/ethnic minority [hazard ratio (HR)=2.30; 95% confidence interval (95% CI): 1.02–5.17] and having systemic symptoms (HR = 2.22; 95% CI: 1.13–4.38) were associated with greater risk of the first positive PCR assay after exposure to a positive IC or HC. Utilizing these bivariable modeling results, we conducted multivariable modeling adjusting for age, race/ethnicity, and sex assigned at birth (Table 2). In our multivariable analysis, we found that a greater risk of a first positive PCR assay was associated with having systemic symptoms (adjusted HR (aHR)=2.62; 95% CI: 1.38–6.55) and identifying as a racial/ethnic minority [aHR = 2.55 for systemic symptoms (95% CI: 1.10–5.94), 2.43 (95% CI: 1.01–5.86) for any respiratory symptoms, and 2.40 (95% CI: 1.03–5.01) for upper respiratory symptoms].

**Table 2:** Multivariable Cox proportional hazard modeling: factors associated with a greater risk of the first positive PCR assay (N = 43).

Characteristic	Any symptoms		Systemic Symptoms		Any respiratory symptoms		Upper respiratory symptoms		Lower respiratory symptoms	
	HR	95% CI	HR	95% CI	HR	95% CI	HR	95% CI	HR	95% CI
**Age in years (continuous)**	0.99	0.98–1.01	1.00	0.98–1.01	1.00	0.98–1.02	1.00	0.98–1.02	1.00	0.98–1.02
**Race**										
Asian	1.5	0.62–3.58	1.64	0.71–3.80	1.98	0.84–4.66	2.02	0.85–4.81	1.92	0.85–4.33
Racial/ethnic minority	2.18	0.94–5.02	**2.55** ^**b**^	**1.10–5.94** ^**b**^	**2.43** ^**b**^	**1.01–5.86** ^**b**^	**2.40** ^**c**^	**1.03–5.61** ^**c**^	2.19	0.93–5.15
White	Ref	Ref	Ref	Ref	Ref	Ref	Ref	Ref	Ref	Ref
**Sex assigned at birth**										
Female	Ref	Ref	Ref	Ref	Ref	Ref	Ref	Ref	Ref	Ref
Male	0.95	0.48–1.86	0.74	0.36–1.54	0.96	0.50–1.85	0.97	0.50–1.86	0.99	0.51–1.92
**Self-reported symptoms on the date of the first positive PCR test?** ^**a**^										
Yes	1.78	0.81–3.89	**2.62** ^**d**^	**1.38–6.55** ^**d**^	0.87	0.42–1.79	0.85	0.40–1.78	1.40	0.44–4.44
No	Ref	Ref	Ref	Ref	Ref	Ref	Ref	Ref	Ref	Ref

^a^**Systemic:** Myalgias (2), fatigue/malaise (10), anosmia (5), ageusia (4), loss of appetite (1), fussiness (in an infant) (1)

**Any Respiratory:** Sore throat (9), rhinorrhea (6), nasal congestion (2), shortness of breath (1), chest pain (1), cough (2)

**Upper respiratory:** Sore throat (9), rhinorrhea (6), nasal congestion (2)

**Lower respiratory:** Shortness of breath (1), chest pain (1), cough (2)

^b^p=0.045

^c^p=0.030

^d^p=0.01

## Discussion

In this study of household transmission of SARS-CoV-2, both symptomatology (systemic symptoms, any respiratory symptoms, and upper respiratory symptoms) and identification as a racial/ethnic minority were associated with a greater risk of a first positive PCR assay after exposure to a positive IC or HC. Previous studies of SARS-CoV-2 Ct values did not collect Ct values longitudinally over extended periods (21 days). We had consistent data using participant-collected nasal swabs, suggesting that this method could be replicated for future studies.

Our findings are consistent with those of previous findings from an international, multi-site study in London (England) and in Portland, Oregon and New York, New York (USA), where anosmia and ageusia were associated with a positive SARS-CoV-2 assay [8]. Although two other studies found no associations between Ct values and symptomatology [8,9], these studies did not evaluate Ct values over time (more than one visit) and they did not include time-to-event analyses – thus limiting the ability to understand the association between Ct values and symptomatology.

In contrast to other studies [6,7], we found that individuals who identified as a racial/ethnic minority had a greater risk of a positive PCR assay when symptomatic (systemic, any respiratory, or upper respiratory symptoms) compared to their white counterparts. However, our findings are consistent with studies that found those who self-identified as African-American and/or Hispanic/Latine had higher SARS-CoV-2 positivity rates compared to people who identified as white [7]. Previous studies found a higher risk of hospitalization, length of stay, ventilation, and death among minorities compared to whites [6,7]. Finally, other studies found that African-American/Latine patients were more likely to seek care at an emergency department due to reduced access to health care [9,10]. Given that our study recruited most of our participants from the emergency department, we may have observed a higher proportion of participants who identified as African-American/Latine for this reason [9,10]. We did not find any correlation between quantitative viral load and symptomatology, which differs from a previous study reporting a statistically significant difference in the mean Ct value in symptomatic vs asymptomatic individuals [11]. Another study during the Omicron wave in early 2022 indicated Ct values peaked approximately four days after symptom onset, which is later than the first positive test [12]. We postulate our results are due to small sample sizes.

The strengths of this analysis included multiple sequential nasal swab samples collected from each participant over 21 days and processed at the same clinical laboratory. Limitations included a small study population (N = 43) with a high chance of type II error because of the limited number of participants [13]. Secondly, the self-reported participant symptomatology was not accompanied by objective assessments by clinicians at the time of RT-PCR testing. Finally, a Ct value is only an approximation of viral load [14].

## Public health implications

In a sizeable geographic setting, we successfully conducted a study where patients self-tested for SARS-CoV-2 using at-home collection kits, and Ct values were analyzed over 21 days. This longitudinal dataset can help us better understand viral load and its association with symptoms and demographic characteristics. This methodology could be used to examine other infectious respiratory diseases, improve clinical guidelines, and reinforce existing public health guidelines such as masking among symptomatic individuals.

In summary, we found that symptomatic patients (specifically, those with systemic symptoms, any respiratory symptoms, and upper respiratory symptoms) and those identifying as a racial/ethnic minority had a greater risk of a positive SARS-CoV-2 PCR assay result after exposure to a positive IC or HC. Future studies should focus on the role of social determinants of health and health disparities on infectious disease transmission, especially in vulnerable households.

## Supporting information

S1 Table Bivariable Cox proportional hazard modeling: factors associated with the first positive PCR assay (N = 43). (DOCX)

S2 Table Sensitivity analysis utilizing Ct cut-off of 34: multivariable Cox proportional hazard modeling examining symptom presence and detectable Ct threshold. (DOCX)

S1 Fig Box and Whisker Plot. Household contact Ct values by study day from enrollment to study end throughout the Household Transmission Study as box and whisker plots among those who ever had a positive SARS-CoV-2 RT-PCR test. N = 43.
(TIFF)
