# Supplementary material for: Associations of SARS-CoV-2 PCR positivity with clinical symptoms and race/ethnicity: The household transmission study
Source: PLoS One. 2025 Sep 30;20(9):e0332819. doi: 10.1371/journal.pone.0332819 (PMC12483199; doi:10.1371/journal.pone.0332819)
Supplement: S1 Table — (DOCX) [file pone.0332819.s001.docx]

# Supporting information

**S1 Table. Bivariable Cox proportional hazard modeling**: factors associated with the first positive PCR assay (N=43)

| Characteristic | HR | 95% CI |
| --- | --- | --- |
| **Age in years (continuous)** | 1.00 | 0.99-1.02 |
| **Race** |  |  |
| White | — | — |
| Asian | 1.87 | 0.84-4.17 |
| Racial/ethnic minority | **2.30^a^** | **1.02-5.17** |
| **Sex assigned at birth** |  |  |
| Male |  |  |
| Female | 1.08 | 0.58-2.02 |
| **Education** |  |  |
| Less than primary school completion | — | — |
| More than primary school, less than secondary school completion | 0.63 | 0.15-2.73 |
| Completed secondary school |  |  |
| College/university/technical college | 1.27 | 0.48-3.34 |
| Missing | 1.42 | 0.46-4.41 |
| **Occupation** |  |  |
| Business/finance | — | — |
| Medicine/healthcare | 1.16 | 0.31-4.33 |
| Missing | 0.54 | 0.18-1.68 |
| Other | 1.89 | 0.36-9.93 |
| Retired | 0.75 | 0.20-2.80 |
| Science/technology | 0.54 | 0.17-1.69 |
| Service/education | 0.61 | 0.19-1.89 |
| **Self-reported symptoms on the date of the first positive PCR test?^b^** |  |  |
| **Any symptoms** |  |  |
| Yes | 1.79 | 0.93-3.44 |
| No |  |  |
| **Systemic symptoms** |  |  |
| Yes | **2.22^c^** | **1.13-4.38 ^c^** |
| No |  |  |
| **Any respiratory symptoms** |  |  |
| Yes | 1.17 | 0.62-2.23 |
| No |  |  |
| **Upper respiratory symptoms** |  |  |
| Yes | 1.10 | 0.56-2.16 |
| No |  |  |
| **Lower respiratory symptoms** |  |  |
| Yes | 1.56 | 0.54-4.49 |
| No |  |  |

^a^ p=0.045

**^b^** Symptoms were coded by matching text in the daily symptom diaries using the regexm command in Stata.

**Systemic:** myalgias (2), fatigue/malaise (10), **anosmia (5), ageusia (4),** loss of appetite (1), fussiness (in an infant) (1)

**Any Respiratory:** sore throat (9), rhinorrhea (6), nasal congestion (2), shortness of breath (1), chest pain (1), cough (2)

**Upper respiratory:**  sore throat (9), rhinorrhea (6), nasal congestion (2)

**Lower respiratory:** shortness of breath (1), chest pain (1), cough (2)

^c^ p=0.02
