# Supplementary material for: Associations of SARS-CoV-2 PCR positivity with clinical symptoms and race/ethnicity: The household transmission study
Source: PLoS One. 2025 Sep 30;20(9):e0332819. doi: 10.1371/journal.pone.0332819 (PMC12483199; doi:10.1371/journal.pone.0332819)
Supplement: S2 Table — (DOCX) [file pone.0332819.s002.docx]

**S2 Table. Sensitivity analysis utilizing Ct cut-off of 34:** multivariable Cox proportional hazard modeling examining symptom presence and detectable Ct threshold

|  | Any symptoms | | Systemic Symptoms | | Any respiratory symptoms | | Upper respiratory symptoms | | Lower respiratory symptoms | |
| --- | --- | --- | --- | --- | --- | --- | --- | --- | --- | --- |
| **Characteristic** | HR | 95% CI | HR | 95% CI | HR | 95% CI | HR | 95% CI | HR | 95% CI |
| **Age in years (continuous)** | 0.99 | 0.96-1.01 | 0.99 | 0.97-1.01 | 0.99 | 0.97-1.01 | 0.99 | 0.97-1.01 | 0.99 | 0.97-1.01 |
| **Race** |  |  |  |  |  |  |  |  |  |  |
| Asian | 1.23 | 0.47-3.27 | 1.32 | 0.51-3.43 | 1.67 | 0.66-4.18 | 1.68 | 0.66-4.26 | 1.76 | 0.71-4.36 |
| Racial/ethnic minority | 1.53 | 0.55-4.20 | 1.59 | 0.58-4.39 | 1.43 | 0.52-3.94 | 1.45 | 0.53-3.94 | 1.37 | 0.49-3.80 |
| White | Ref | Ref | Ref | Ref | Ref | Ref | Ref | Ref | Ref | Ref |
| **Sex assigned at birth** |  |  |  |  |  |  |  |  |  |  |
| Female | Ref | Ref | Ref | Ref | Ref | Ref | Ref | Ref | Ref | Ref |
| Male | 0.66 | 0.30-1.45 | 0.51 | 0.22-1.21 | 0.69 | 0.32-1.50 | 0.69 | 0.32-1.50 | 0.72 | 0.33-1.57 |
| **Self-reported symptoms on the date of the first positive PCR test?^a^** |  |  |  |  |  |  |  |  |  |  |
| Yes | 2.41 | 0.96-6.05 | **3.01^b^** | **1.25-7.26^b^** | 1.06 | 0.47-2.40 | 1.02 | 0.43-2.42 | 1.59 | 0.42-5.98 |
| No | Ref | Ref | Ref | Ref | Ref | Ref | Ref | Ref | Ref | Ref |

^a^ Symptoms were coded by matching text in the daily symptom diaries using the regexm command in Stata.

**Systemic:** myalgias (2), fatigue/malaise (10), **anosmia (5), ageusia (4),** loss of appetite (1), fussiness (in an infant) (1)

**Any Respiratory:** sore throat (9), rhinorrhea (6), nasal congestion (2), shortness of breath (1), chest pain (1), cough (2)

**Upper respiratory:**  sore throat (9), rhinorrhea (6), nasal congestion (2)

**Lower respiratory:** shortness of breath (1), chest pain (1), cough (2)

^b^ p=0.01
