# Supplementary figures and images for: Associations of SARS-CoV-2 PCR positivity with clinical symptoms and race/ethnicity: The household transmission study
Source: PLoS One. 2025 Sep 30;20(9):e0332819. doi: 10.1371/journal.pone.0332819 (PMC12483199; doi:10.1371/journal.pone.0332819)

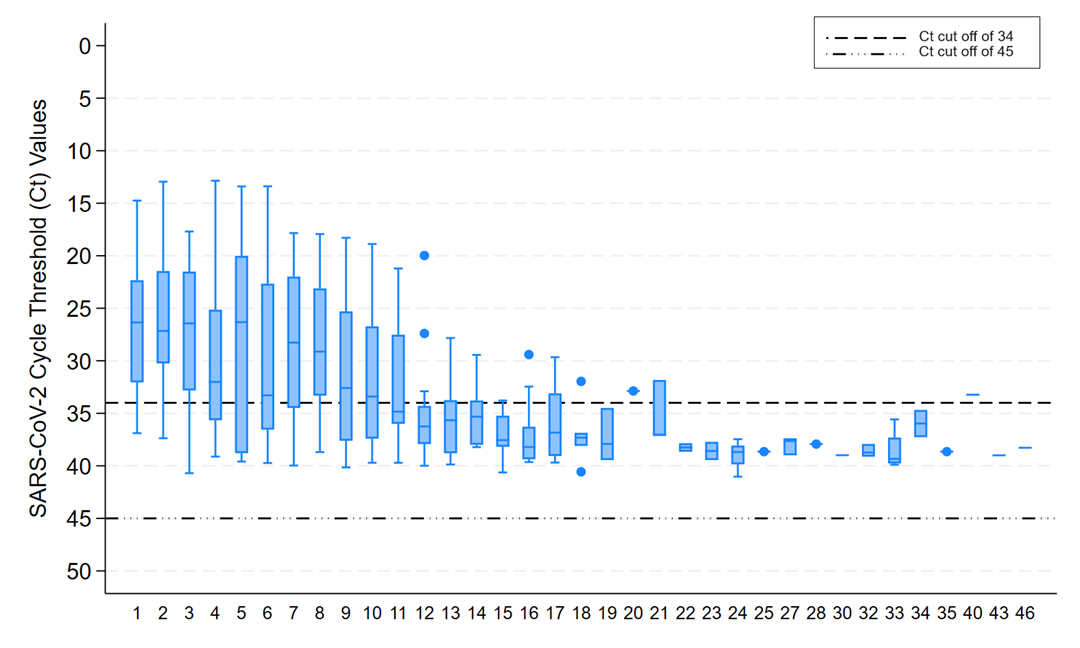

Supplement: S1 Fig — Household contact Ct values by study day from enrollment to study end throughout the Household Transmission Study as box and whisker plots among those who ever had a positive SARS-CoV-2 RT-PCR test. N = 43. (TIFF) [file pone.0332819.s003.tiff]
